# Monthly Summary

**Published:** 1893-08

**Authors:** 


### Monthly Summary.

THE USE OF ANÆSTHETICS IN EXTRACTING TEETH.—Few minor operations are more dreaded by people generally than the extraction of teeth. Indeed, it has been so from the beginning of the world, and from the cradle to the grave. Yet it is something that almost every one is compelled to undergo at some time in life, and some unfortunate ones have the operation repeated many times.

It has been questioned whether one is justified in administering a general anæsthetic to relieve the pain and dread of this unpleasant operation. The late Dr. W. H. Atkinson was very decided in his views on this subject, and condemned severely a practice that would put a human being within one step of death's door for so slight an operation. As members of a progressive profession it becomes our duty to remove as much of the barbarous feature as possible, thereby relieving as much of the dread of dental operations as we can by the use of both local and general anæsthetics.

There is no reason why we should not *progress* in this direction as well as any other. We have the drugs; let us learn to use them for the convenience and comfort of our patients. Once in a while, it is true, we see an article stating that death has been produced from inhaling nitrous oxide gas or from an injection of cocaine, or from the use of some other of our most harmless alleviators of pain. Most of these notices come from newspaper reports, the accuracy of which is always questionable, and before we condemn too severely these old tried friends we should investigate the case thoroughly. My sentiments are somewhat voiced in an editorial clipped from the *Brooklyn Medical Journal*, as follows:

"A Buffalo lady who had gone to a dentist to have some teeth extracted, is reported to have died in the chair while inhaling nitrous oxide gas. If this report is correct we must noc attribute the death to the gas until we know more fully the history of the patient. Of all the anæsthetics that we have at our disposal, none is so safe as nitrous oxide; indeed, we have come to believe that it is absolutely devoid of danger. The enormous number of instances in which it has been safely administered certainly justifies this opinion."

I do not wish to be understood as advising or recommending the use of anæsthetics for every little trivial operation, but we should be prepared, competent and accomplished in their administration when necessary.

When roots are to be removed, abscessing or dead teeth, the pain of the operation can be almost entirely removed by an injection of a weak solution of cocaine in the tissues surrounding. A strong solution, nor a great quantity is not necessary, and the relief very perceptible. If a separator is to be applied, a gold band to be fitted, a strong medicine to be injected, the pain can be almost entirely removed by the judicious use of this wonderful medicine. These little details in practice should be looked after. If it takes time, give it and charge for it. You will rarely have patients object to paying for extra time and trouble, if by so doing they can be relieved of pain and inconvinience. Work on this line. Go slowly and carefully and watch the results.—EDITORIAL in *Southern Dental Journal*.

---

CEMENTS AS ROOT FILLINGS.—The zinc cements, both oxyphosphate and chloride, have for some years probably been the most popular materials for root filling in devitalized teeth that we have. Their ease of manipulation and supposed insolubility when protected from the fluids of the mouth, largely account for this. At one time I strongly advocated the chloride on account of its being generally slow setting, and possessing antiseptic qualities. Finding objections to this, I tried the oxyphosphate, which, being of a harder and more durable mass when set, is, in many respects, superior, but itself not free from objections.

We all see devitalized teeth that have been filled for years without giving any inconvenience whatever, when suddenly the patient is made known of its presence by the sudden development of a severe case of pericementitis. This often is a puzzle to both patient and operator. By careful observation and experience in my own practice, in the use of these materials, I have come to the conclusion that these condition are not uncommon where they have been used. Hardly any cement filling in the root of a tooth, with an apical foramen large enough to admit fluids, will remain longer than a year or two without absorbing enough poisonous product of decomposition to give off a perceptible odor. Of the two, the chloride is the less durable, though I do not consider either at all a desirable material for root filling, unless the apical foramen has been well sealed with some more indestructible material before its insertion. A tooth that is filled with it may be expected to give trouble sooner or later, and it must soon be discarded altogether and give place to some more durable and desirable material.—EDITORIAL in *Southern Dental Journal.*

---

IN USING COCAINE, remember the following propositions:

"1. Cocaine may be toxic.

"2. This effect is not rare.

"3. There is a lethal dose of cocaine.

"4. This dose is uncertain.

"5. Dangerous or deadly results may follow doses usually deemed safe.

"6. Toxic effects may be the sequence of doses large or small, in patients young or old, the feeble or the strong.

"7. The danger, near and remote, is greatest when given under the skin.

"8. Cardiac or renal weakness increases the risk.

"9. Purity of drug will not exempt from ill result.

"10. Caution is needful under all conditions."—"Cosmos.'

---

PHILOSOPHERS AND CRANKS are prone to classify mankind on opposite sides of the line of their particular hobby. Swift divided the human race into two classes—the fat and lean. Our dominie thinks they should be classified as those who believe in the true religion and unbelievers. Old Moneygrub arranges them as the rich and the poor; while our friend Politix says the only two classes are the inns and the outs. At the last judgment we are all to be classified as the sheep and the goats.

NEW METHOD OF ADJUSTING LOGAN CROWNS.—The desirability of the Logan crown as to strength and anatomical form is very generally recognized, and the following method obviates the difficulty of obtaining a perfect joint between the stump of the root and the crown. After sealing the apical opening, grind the root under the free margin of the gum, select the proper teeth, wrap very thin platina plate once around the entire length of the pin and solder with pure gold, then burnish this barrell about the pin. Now prepare the root to receive this barrell, then burnish 32 gauge pure gold over the stump, solder the barrel to the cap, insert barrel and cap, and with a wooden dowel mallet and burnish cap to a perfect joint with the stump and trim the cap to the periphery.

Now remove cap and barrell, and you have the exact surface of the stump *out of the mouth*, to which you can rapidly grind the tooth to fit. Then cement the barrel and cap to place on the root. We then have the root perfectly protected and a metal wall perfectly fitted to the post and crown. When cement is hard, then insert crown to place. The advantages in this new method may be summarized as follows:

1. That the difficulty of making a good joint in the mouth is overcome.

2. The stump is perfectly protected.

3. The tooth with post when finally inserted fits tightly against metal surfaces, only a minimum amount of cement being required.

4. Any dislodging force must bear on every surface of both post and stump.—EDITORIAL in *Western Dental Journal.*

---

PRESENCE OF MIND IN APPLYING AN ANTIDOTE.—An instance of rare presence of mind attended by success in the use of an antidote to poisoning occurred recently at Sag Harbor, N. Y.

Flora Sterling, the five-year-old daughter of Dr. Sterling, while playing about the house found a bottle which had formerly contained citrate of magnesia, and still bore the label. The child took a long swallow. With a scream she dropped the bottle and began to clutch her little throat in an agony of pain. Her father, who had heard her screams, found that what the little one had taken for citrate of magnesia was oxalic acid. Seeing that not a moment was to be lost, if he wished to save the child's life, the doctor looked about for an alkaline antidote. Seizing his pen-knife the doctor sprang to the white-washed wall and scraped some of the lime into his hand. This he threw into a glass partly filled with water, and poured the mixture down the atmost dying child's throat. The antidote took effect at once. The intense pain caused by the burning acid was alleviated, and soothing, mucilaginous drinks to cool the blistered mouth and throat did the rest.—*Scientific American.*

---

DR. WM. CRENSHAW, Atlanta, Ga., made a four crown gold bridge for Dr. W. L. Jones, of Jackson, Miss. The abutments were the first right lower bicuspid and third molar. Evans' seamless gold crowns were used in its construction. After fitting the abutment crowns to the first bicuspid and the third molar and properly articulating them, a bite was obtained and an impression taken with the crowns in position. From this impression a model was made as usual. The case was next placed in an articulator and an opposing model made from the bite. After this two Evans' crowns were selected, suitable to fill the gap to be bridged. These were sloped off from the under side, filled with solder, waxed in place, properly adjusted, invested and soldered.

Before investing, the dummy crowns were taken out of the articulator and placed in the mouth to see that the bite was correct. A piece of wax placed under the crowns will prevent them being displaced when the teeth close on them in testing the bite.—*Southern Dental Journal.*

